# Disability for basic and instrumental activities of daily living in older individuals

**DOI:** 10.1371/journal.pone.0220157

**Published:** 2019-07-26

**Authors:** Juan Manuel Carmona-Torres, María Aurora Rodríguez-Borrego, José Alberto Laredo-Aguilera, Pablo Jesús López-Soto, Esmeralda Santacruz-Salas, Ana Isabel Cobo-Cuenca

**Affiliations:** 1 Facultad de Fisioterapia y Enfermería de Toledo, Universidad de Castilla-La Mancha (UCLM), Toledo, Spain; 2 Grupo de Investigación Multidisciplinar en Cuidados (IMCU), Universidad de Castilla la Mancha, Toledo, Spain; 3 Instituto Maimónides de Investigación Biomédica de Córdoba (IMIBIC), Córdoba, Spain; 4 Universidad de Córdoba (UCO), Córdoba, Spain; 5 Hospital Universitario Reina Sofía de Córdoba (HURS), Córdoba, Spain; 6 Facultad de Ciencias de la Salud, Universidad de Castilla-La Mancha (UCLM), Talavera de la Reina, Spain; UCIBIO-REQUIMTE, Faculty of Pharmacy, University of Porto, PORTUGAL

## Abstract

**Aims:**

To know the prevalence, associated factors and temporal trends of disabilities for basic and instrumental activities of daily living in older people in Spain from 2009 to 2017.

**Background:**

Disability in older people is associated with health problems, increased health costs and low quality of life. There are no updated data in Spain with a representative sample about disability.

**Methods:**

Cross-sectional study with 25,465 non-institutionalized older people who participated in the European Health Survey in 2009 and 2014 and the National Health Survey in 2011/12 and 2017 in Spain. The prevalence rates of disability were evaluated using the Katz Scale and Lawton and Brody Scale. Logistic regression was used to determine if there was an association between basic and instrumental activities of daily living and sociodemographic characteristics.

**Results:**

More individuals had disability for instrumental activities of daily living (31.9%) than disability for basic activities of daily living (11.1%). The most predominant disability for instrumental activities of daily living was performing severe housework (34%). The prevalence of disabilities decreased from 2009 to 2017. In general, disability was associated with female gender, advanced age, lower education, restricted daily activity, being bedridden and higher pain levels.

**Conclusion:**

There is a considerable prevalence of disabilities for basic and instrumental activities of daily living in older people in Spain. Although the disability prevalence has decreased slowly from 2009 to 2017, it continues to remain a health problem. Gender may influence the disabilities for basic and instrumental activities of daily living. Health policymakers should establish prevention strategies and effective interventions (e.g., physical exercise) for prevention and reduction of the disabilities for basic and instrumental activities of daily living, particularly in older females.

## Introduction

The world's population is an increasingly aging population [1]. Spain, in particular, is aging at one of the highest rates in Europe and the world. It is estimated that by 2050, the population over 60 years old will reach 41.4% of the total population in Spain [2]. Along with increasing age in recent decades, there have been increased numbers of individuals who have chronic disorders, disabilities and functional limitations [3]. Characteristics of aging can also lead to vulnerability, disability and increased frailty [4].

Older people also may have decreased functional capacity due to alterations in physiology during their lifespan; as a result, they demonstrate disabilities in the Basic Activities of Daily Living (ADL) and Instrumental Activities of Daily Living (IADL) [5]. The ADL are fundamental activities for independent life at home, which include bathing, feeding oneself, etc. The IADL are more complex activities that require a higher level of autonomy and cognitive function and are necessary for independent life in the community [6,7].

Functional capacity can be defined as the potential of elderly people to decide and act independently in their daily living [8]; disability can be defined as the difficulty or need for help to perform daily activities [8].

Disability in the elderly is particularly measured by the capacity for individuals to carry out ADL and IADL [7,9]. When there is greater dependency on others for ADL and IADL, the disability is considered more severe [10]. Disability leads to increased illness, which can produce further disability and dependence on others [11,12].

Healthy aging correlates with good performance of the ADL and IADL [6]. Therefore, it is speculated that improving ADL and IADL may assist in preventing disability in this population [13]. In addition, nurses can help prevent or remedy this disability for ADL and IADL.

Age likewise is associated with the major probability of having a disability; this can have repercussions in health and social systems [14], because ADL and IADL can increase the necessity of personal assistance [7].

Studies from the United States [15] and Sweden [16] have analyzed the temporal trends of functional dependence. These studies showed that disability remained steady in time with a gradual tendency for performance to decline. Previous studies in Spain showed that increased disability was associated with being female, being older, having lower educational levels and being obese [17–19]. However, there are no updated studies that analyze the temporal trends of functional dependence in non-institutionalized older people in Spain.

The aim of this study was to estimate the prevalence of disabilities for ADL and IADL in people over 65 years old in Spain, the associated factors for disability, and the temporal trends for functional dependence from 2009 to 2017.

## Materials and methods

### Design and participants

We conducted a cross-sectional analysis using publicly available sample survey data. Information sources were microdata from the European Health Survey in Spain (EHSS) 2009 [20] and 2014 [21] and the National Health Survey in Spain (NHSS) 2011/12 [22] and 2017 [23]. These surveys were performed by the National Health Institute (NHI) of Spain and the Ministry of Health, Social Services and Equality (MHSSE) of Spain. EHSS and NHSS were completed by a non-institutionalized Spanish population through personalized interviews. According to Spanish legislation, because we used public anonymized secondary data, approval by an ethics committee was not necessary.

EHSS and NHSS utilized a probabilistic multi-stage sampling method with stratification of the first- and second-stage units. The units of the first-stage were the census section, the units of the second-stage were the main family dwellings, and the units of the third-stage were individuals who were present in the household; those individuals were selected by random sampling and quotas based on sex and age. Each participant was assigned a weighted coefficient to ensure the representativeness of the sample.

Microdata were anonymized and are available on the NHI of Spain website [20–23]. Microdata contained the records of Spanish participants over 15 years old. This study looked at individuals 65 years old and older. The total number of records was 25,465: 6,026 in 2009, 5,896 in 20011/12, 6,520 in 2014 and 7,023 in 2017.

### Variables

We utilized the following sociodemographic variables (contained in each record): age, gender, nationality, cohabitation with a partner, marital status, educational level, pension contribution, self-perceived health status in the prior 12 months, chronic diseases, restriction of habitual activity during the past 2 weeks, bedridden status during the past 2 weeks, use of glasses or hearing aids, difficulty seeing or hearing, difficulty walking 500 meters or up 12 stairs, pain during the past 4 weeks, social class and body mass index (BMI).

The main variable in this study was disability, which included ADL and IADL. ADL independence was assessed using five items based on the Katz Scale [24] (feeding oneself, sitting down and getting up from a bed and chair by oneself, dressing and undressing, using a toilet and bathing). The Katz Scale has good validity and reliability greater than 0.90. Participants were considered to have an ADL disability if they answered “*I am unable to do it by myself*” or “*I have a lot of difficulty*” for one or more items. IADL independence was assessed using seven items based on the Lawton and Brody Scale [25] (preparing own meals, using the telephone, going shopping, taking medications, performing light housework, performing severe housework and managing money). The Lawton and Brody Scale has validity of 0.85 and reliability between 0.87 and 0.91. Participants were considered to have an IADL disability if they answered *“I cannot do it at all*” or *“I have a lot of difficulty”* for one or more items. These classifications for disability have been used in previous studies [1,9,16,19].

### Statistical analysis

Descriptive measures for all variables of interest were used, utilizing count (n), percentage (%), mean (m) and standard deviation (SD). The chi-square test (χ^2^) was used to compare categorical variables between groups. Logistic regression analyses were used to estimate the association of the independent variables with ADL disability and IADL disability. The Wald test was used, and the variables for which p≥0.15 were eliminated one by one from the model. The results are presented as odds ratios (ORs) and their respective 95% confidence intervals (95% CI). Statistical significance was established at p<0.05 (2-tailed *p* values).

Data were analyzed using IBM SPSS Statistic v.24.0 (IBM Corp., Armonk, NY, USA) licensed by the University of Castilla La-Mancha.

## Results

The total sample was 25,465 individuals over 65 years old, with a mean age of 75.85 years (SD±7.44). Of them, 60.6% were women and 39.4% were men. Although the total sample lived in Spain, only 98.7% had Spanish nationality. Individuals were most frequently married (49.6%), had a primary or secondary educational level (46.6%), had a pension contribution (95.8%), had a good self-perceived health status (37%), had a chronic disease (84.7%), did not have difficulty walking 500 meters (69.2%), did not have difficulty going up or down 12 stairs (59.9%), had pain in the past 4 weeks (61.1%), belonged to the V social class (skilled workers in the primary sector and other semi-skilled workers) (35.6%) and were overweight (39.1%). The majority (85.9%) of older people used glasses or contact lenses, although the majority had no difficulty seeing (78.9%). Only 8.5% were using hearing aids, but 26.7% had difficulty hearing. Demographic characteristics are shown in Table 1.

**Table 1:** Sociodemographic characteristics of Spanish people over 65 years old (2009–2017) (n = 25,465).

Sociodemographic characteristics	2009 (n = 6,026)	2011/12 (n = 5,896)	2014 (n = 6,520)	2017 (n = 7,023)
*Sex*				
Male	2,330 (38.7%)	2,223 (37.7%)	2,624 (40.2%)	2,850 (40.6%)
Female	3,696 (61.3%)	3,673 (62.3%)	3,896 (59.8%)	4,173 (59.4%)
*Age*				
65–74 years	2,849 (47.3%)	2,731 (46.3%)	3,125 (47.9%)	3,383 (48.2%)
75–84 years	2,426 (40.3%)	2,350 (39.9%)	2,423 (37.2%)	2,543 (36.2%)
≥ 85 years	751 (12.5%)	815 (13.8%)	972 (14.9%)	1,097 (15.6%)
*Marital status*				
Single	536 (8.9%)	489 (8.3%)	554 (8.5%)	572 (8.1%)
Married	2,926 (48.6%)	2,912 (49.4%)	3,234 (49.6%)	3,567 (50.8%)
Widowed	2,396 (39.8%)	2,310 (39.2%)	2,502 (38.4%)	2,567 (36.6%)
Separated/Divorced	163 (2.7%)	180 (3.1%)	224 (3.4%)	133 (4.4%)
*Nationality*				
Spanish	5,943 (98.6%)	5,827 (98.8%)	6,442 (98.8%)	6,924 (98.6%)
Other	83 (1.4%)	69 (1.2%)	78 (1.2%)	99 (1.4%)
*Educational level*				
Without education	2,805 (46.6%)	Not registered	2,230 (34.2%)	2,136 (30.4%)
Primary or secondary	2,376 (39.5%)		3,209 (49.2%)	3,522 (50.1%)
Professional training	478 (7.9%)		564 (8.7%)	730 (10.4%)
University	355 (5.9%)		517 (7.9%)	635 (9.0%)
*Self-perceived health status*				
Very good	298 (4.9%)	359 (6.1%)	422 (5.5%)	411 (6.3%)
Good	2,042 (33.9%)	2,205 (37.4%)	2,428 (37.2%)	2,745 (39.1%)
Regular	2,285 (37.9%)	2,175 (36.9%)	2,371 (36.4%)	2,593 (36.9%)
Bad	1,029 (17.1%)	915 (15.5%)	955 (14.6%)	977 (13.9%)
Very bad	372 (6.2%)	242 (4.1%)	344 (5.3)	267 (3.8%)
Chronic diseases				
Yes	5,028 (83.4%)	4,302 (73.0%)	5,750 (88.2%)	6,493 (92.5%)
No	993 (16.5%)	1,589 (27.0%)	769 (11.8%)	527 (7.5%)
Not responding	5 (0.1%)	5 (0,1%)	1 (0.0%)	3 (0.0%)
Restriction of habitual activity*				
Yes	Not registered	952 (16.1%)	1,351 (20.7%)	1,294 (18.4%)
No		4,944 (83.9%)	5,169 (79.3%)	5,729 (81.6%)
*Bedridden*^†^				
Yes	Not registered	418 (7.1%)	531 (8.2%)	519 (7.4%)
No		5,478 (92.9%)	5,987 (91.8%)	6,504 (92.6%)
*Use of glasses*				
Yes	5,224 (86.7%)	5,102 (86.5%)	5,516 (84.6%)	6,027 (85.8%)
No	767 (12.7%)	771 (13.1%)	987 (15.1%)	980 (14.0%)
I am blind or I cannot see at all	33 (0.5%)	22 (0.4%)	17 (0.3%)	16 (0.2%)
*Use of hearing aids*				
Yes	452 (7.5%)	489 (8.3%)	593 (9.1%)	629 (9.0%)
No	5,555 (92.2%)	5,387 (91.4%)	5.913 (90.7%)	6,378 (90.8%)
I am deaf	17 (0.3%)	17 (0.3%)	13 (0.2%)	16 (0.2%)
*Difficulty walking 500 meters*				
No, without difficulty	3,870 (64.2%)	5,060 (85.8%)	4,181 (64.1%)	4,510 (64.2%)
Yes, some difficulty	957 (15.9%)	544 (9.2%)	1,132 (17.4%)	1,246 (17.7%)
Yes, a lot of difficulty	490 (8.1%)	0	804 (12.3%)	786 (11.2%)
I cannot do it at all	708 (11.7%)	289 (4.9%)	402 (6.2%)	481 (6.8%)
*Difficulty going up or down 12 stairs*				
No, without difficulty	3,046 (50.5%)	4,512 (76.5%)	3,728 (57.2%)	3,926 (56.4%)
Yes, some difficulty	1,374 (22.8%)	678 (11.5%)	1,341 (20.6%)	1,479 (21.1%)
Yes, a lot of difficulty	626 (10.4%)	0	969 (14.9%)	1,041 (14.8%)
I cannot do it at all	976 (16.2%)	696 (11.8%)	480 (7.4%)	543 (7.7%)
*Pain during the past four weeks*				
Yes	3,286 (54.7)	Not registered	4,131 (63.5%)	4,529 (64.5%)
No	2,725 (45.3%)		2,378 (36.5%)	2,489 (35.5%)
*Body Mass Index*				
Underweight (<18.5) or Normal Weight (18.5–25)	1,546 (25.7%)	1,417 (24.0%)	1,898 (29.1%)	72 (29.2%)
Overweight (25–30)	2,361 (39.2%)	2,116 (35.9%)	2,644 (40.6%)	2,841 (40.5%)
Obesity (>30)	1,210 (20.1%)	1,158 (19.6%)	1,369 (21%)	1,487 (21.2%)
Not responding	909 (15.1%)	1,205 (20.4%)	609 (9.3%)	645 (9.2%)

* During the past two weeks

A minority (11.1%) had an ADL disability, with a mean number of 2.69 (SD±1.54). ADL with the most disabilities were bathing (9.9%) and dressing/undressing (6.6%). More subjects had an IADL disability (31.9%), with a mean number of 2.88 (SD±2.13), as compared to subjects who had an ADL disability. The largest categories of IADL disabilities were performing severe housework (34%), performing light housework (16.6%) and going shopping (14.6%). Table 2 shows the disabilities for each item of the ADL and IADL.

**Table 2:** Disability for ADL and IADL of Spanish people over 65 years analyzed by items (2009–2017).

	2009 n = 6,026	2011/12 n = 5,896	2014 n = 6,520	2017 n = 7,023
*Disability for ADL*				
Feeding oneself	189 (3.2%)	276 (4.7%)	168 (2.6%)	180 (2.6%)
Sitting down and getting up from a bed and chair by oneself	436 (7.3%)	268 (4.5%)	357 (5.5%)	426 (6.1%)
Dressing and undressing	486 (8.1%)	304 (5.2%)	413 (6.3%)	480 (6.9%)
Using a toilet	349 (5.8%)	Not registered	340 (5.2%)	395 (5.7%)
Bathing	694 (11.5%)	415 (7%)	674 (10.4%)	727 (10.4%)
*Disability for IADL*				
Preparing own meals	921 (15.3%)	564 (9.6%)	654 (10%)	655 (9.3%)
Using the telephone	487 (8.1%)	364 (6.2%)	505 (7.7%)	489 (7%)
Going to shopping	1,018 (16.9%)	718 (12.2%)	1,001 (15.4%)	975 (13.9%)
Taking medications	494 (8.2%)	360 (6.1%)	504 (7.7%)	488 (6.9%)
Performing light housework	1,193 (19.8%)	923 (15.7%)	1,035 (15.8%)	1,062 (15.2%)
Performing severe housework	2,221 (36.9%)	Not registered	2,088 (32%)	2,345 (33.4)
Managing money	639 (10.6%)	449 (7.6%)	631 (9.7%)	590 (8.4%)

ADL = Basic activities of daily living; IADL = Instrumental activities of daily living

**Fig 1:**
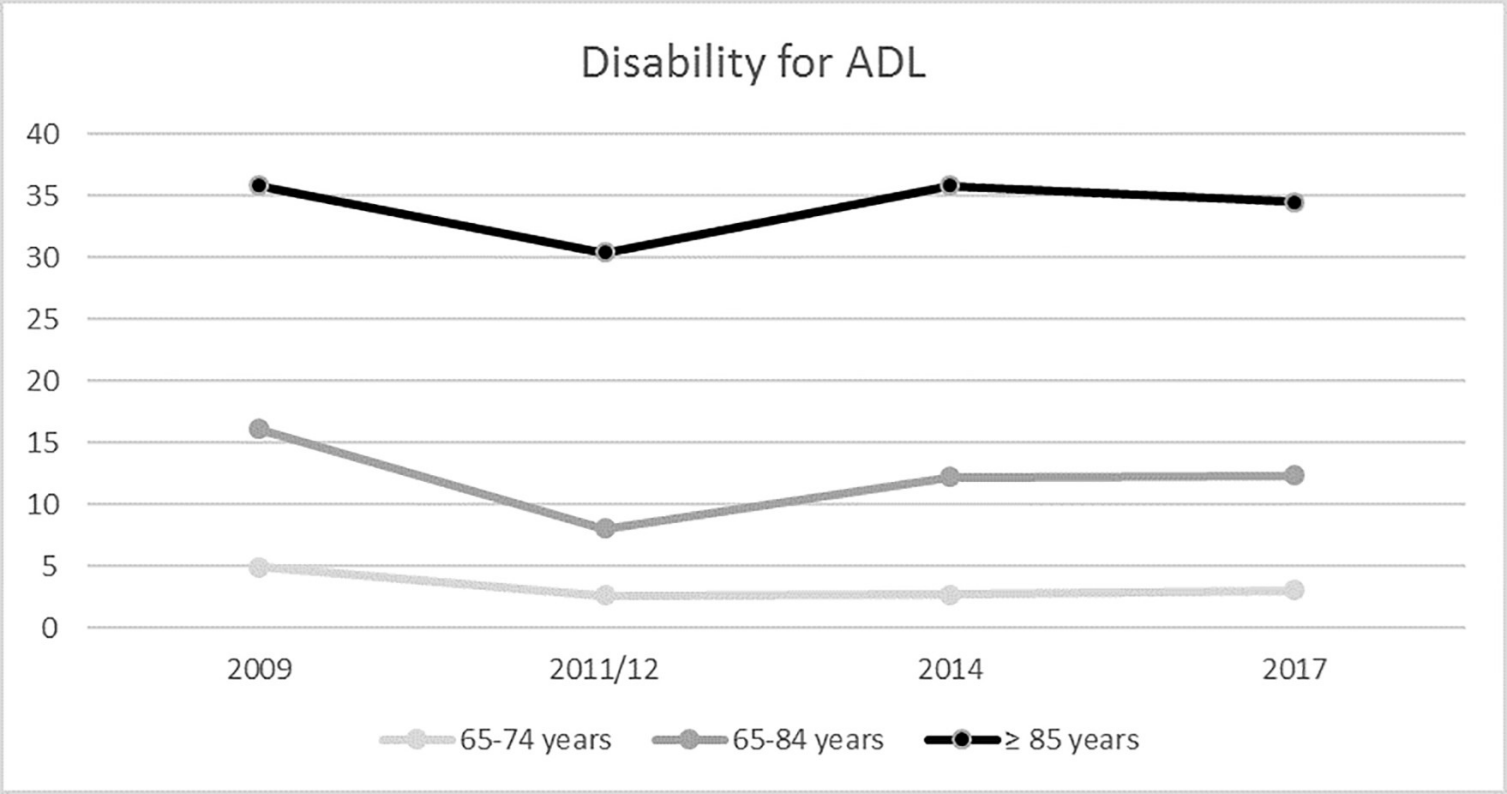
Disability for ADL in Spanish people over 65 years old analyzed by age group (2009–2017).

**Fig 2:**
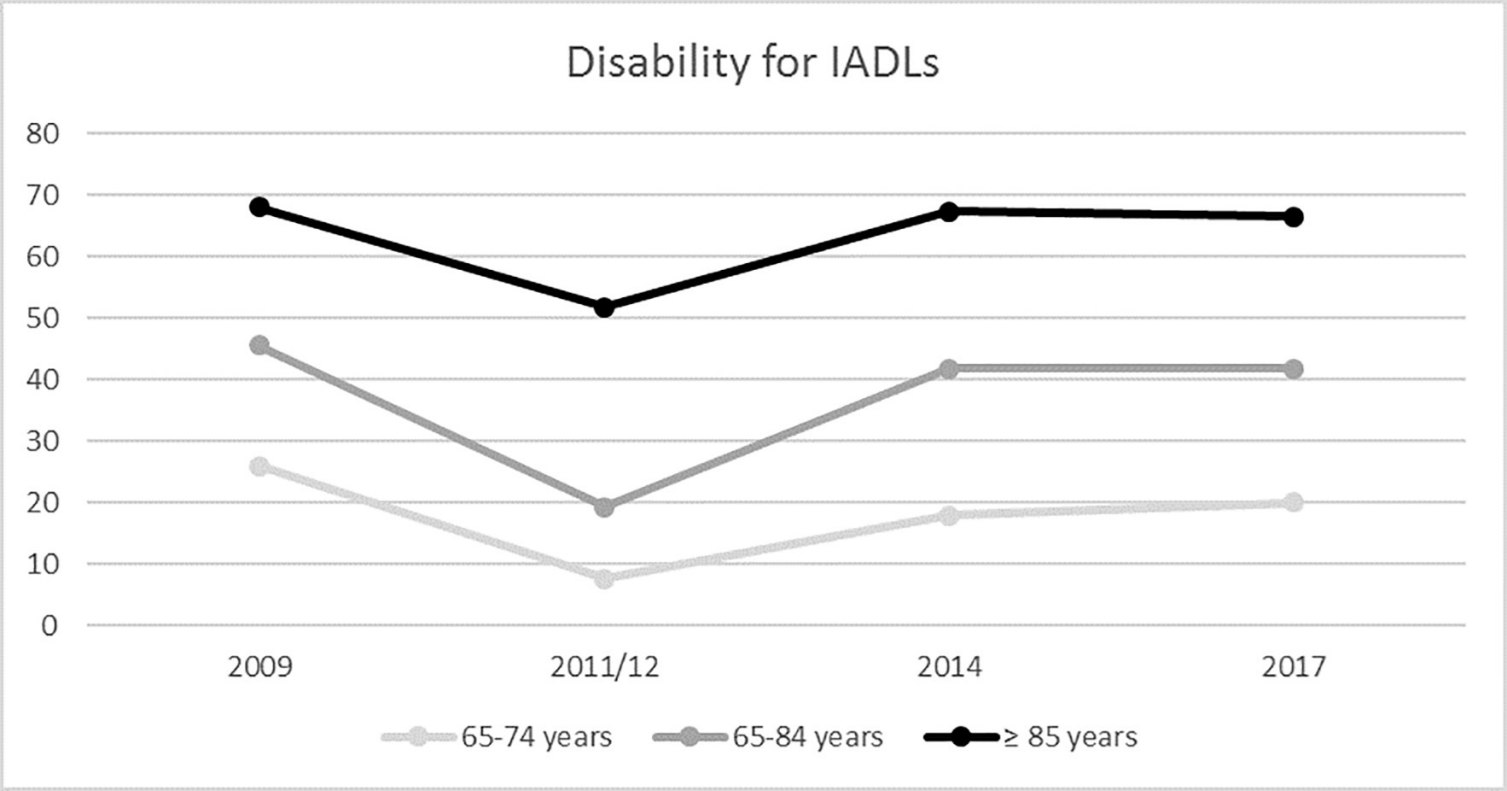
Disability for IADL in Spanish people over 65 years old analyzed by age group (2009–2017).

**Fig 3:**
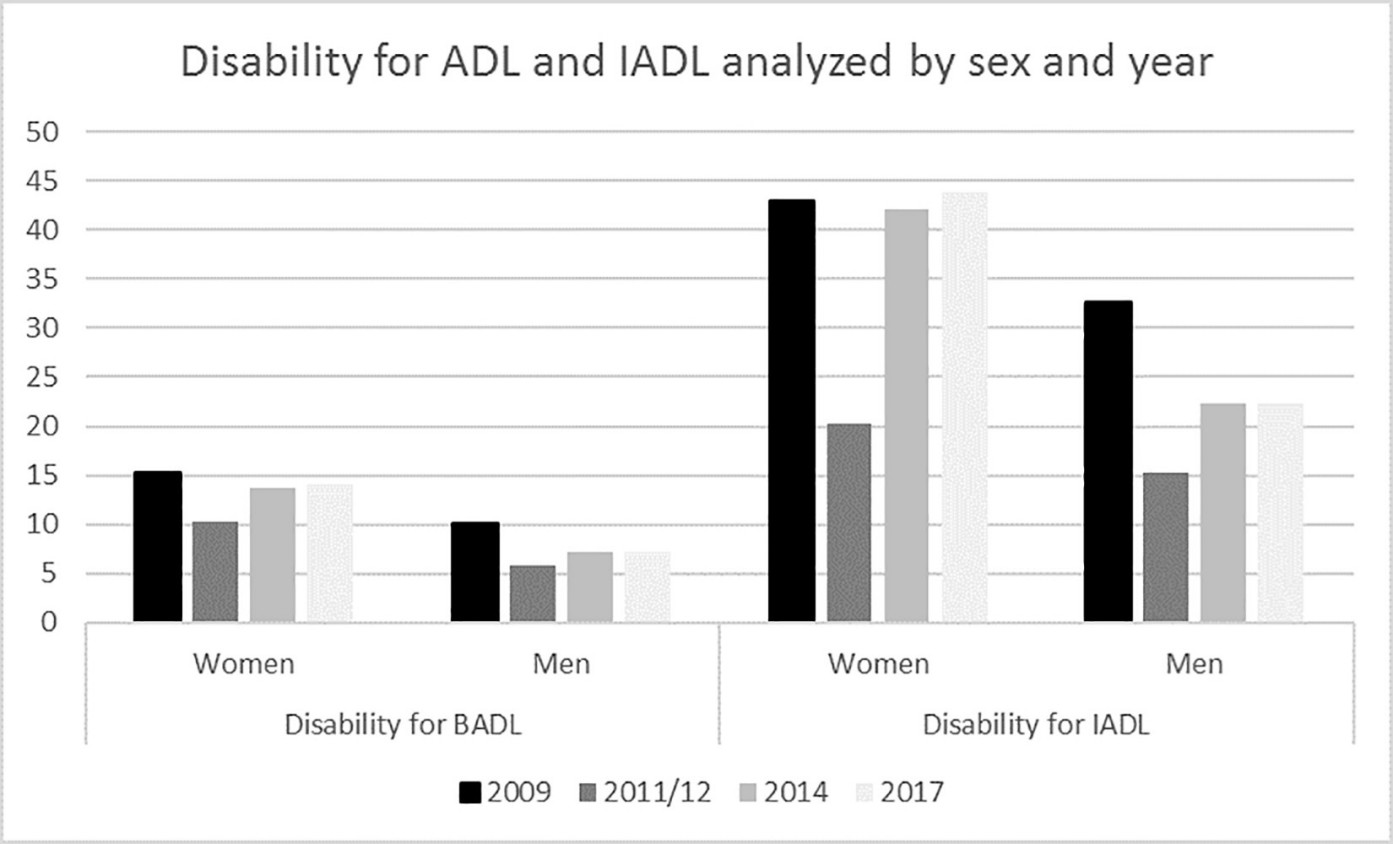
Disabilities for ADL and IADL in Spanish people over 65 years old analyzed by sex and year (2009–2017).

Figs 1 and 2 show temporal trends for ADL and IADL disabilities in elderly individuals. ADL disability decreased from 13.2% in 2009 to 11.3% in 2017 (p<0.001). IADL disability decreased from 39% in 2009 to 35.1% in 2017 (p<0.001). ADL and IADL disabilities were analyzed by sex and year (Fig 3). Women had greater numbers of ADL and IADL disabilities than that of men in all years of the study (p < 0.001).

The ≥ 85 years old age group demonstrated more ADL and IADL disabilities than that of the other age groups (65–74 years old and 75–84 years old) (Figs 1 and 2). ADL disability decreased from 2009 to 2017 in all age groups (p<0.001). IADL disability also decreased from 2009 to 2017 in all age groups (p<0.001). ADL and IADL disabilities decreased from 2009 to 2011/12, but they later increased slightly until 2017.

The logistic regression analysis (Table 3) showed that ADL disability was independently associated with women (OR 1.39, 95% CI 1.21–1.6, p<0.001), age older than 85 years (OR 15.76, 95% CI 13.08–18.99, p<0.001), lower educational status (OR 2.7, 95% CI 1.91–3.8, p<0.001), having habitual activity restrictions (OR 3.22, 95% CI 2.77–3.74, p<0.001), being bedridden during the past 2 weeks (OR 3.04, 95% CI 2.52–3.65, p<0.001) and having pain in the past 4 weeks (OR 1.88, 95% CI 1.58–2.24, p<0.001).

**Table 3:** Logistic Regression Model for the association among sociodemographic characteristics and disability for ADL and IADL in Spanish people over 65 years (2009–2017) (n = 25,465).

	ADL Disability		IADL Disability	
	OR (95% CI)	p	OR (95% CI)	p
*Sex*				
Men	*Reference*		*Reference*	
Women	1.39 (1.21–1.6)	<0.001	1.93 (1.76–2.11)	<0.001
*Age Group*				
65–74 years	*Reference*		*Reference*	
75–84 years	3.88 (3.25–4.65)	<0.001	2.58 (2.35–2.83)	<0.001
≥ 85 years	15.76 (13.08–18.99)	<0.001	7.03 (6.22–7.95)	<0.001
*Level of education*				
Without education	2.7 (1.91–3.8)	<0.001	2.63 (2.19–3.18)	<0.001
Primary or secondary	1.54 (1.06–2.17)	0.013	1.5 (1.25–1.8)	<0.001
Professional training	1.11 (0.72–1.72)	0.628	1.17 (0.94–1.48)	0.165
University	*Reference*		*Reference*	
Restriction of habitual activity*				
Yes	3.22 (2.77–3.74)	<0.001	2.65 (2.36–2.97)	<0.001
No	*Reference*		*Reference*	
*Bedridden*^†^				
Yes	3.04 (2.52–3.65)	<0.001	1.87 (1.58–2.22)	<0.001
No	*Reference*		*Reference*	
*Pain during the past four weeks*				
Yes	1.88 (1.58–2.24)	<0.001	2.8 (2.53–3.09)	<0.001
No	*Reference*		*Reference*	

OR = odds ratio; CI = confidence interval

*during the past two weeks.

The logistic regression analysis (Table 3) showed that IADL disability was independently associated with women (OR 1.93, 95% CI 1.76–2.11, p<0.001), age older than 85 years (OR 7.03, 95% CI 6.22–7.95, p<0.001), lower educational level (OR 2.63 95% CI 2.19–3.18, p<0.001), restriction of habitual activity (OR 2.65, 95% CI 2.36–2.97, p<0.001), bedridden during the past 2 weeks (OR 1.87, 95% CI 1.58–2.22, p<0.001) and pain in the past 4 weeks (OR 2.8, 95% CI 2.53–3.09, p<0.001).

## Discussion

Disability can be either a result of physiopathology and/or biological alterations in older individuals. It is an important health issue as society ages. From a general point of view, incapacity for performing the activities of daily living in older people decreases their quality of life, increases sanitary costs and contributes to premature death [26–29]. Recent studies show that requiring major dependence on others for ADL is a risk factor for elder abuse [30]. These factors contribute to increasing frailty and vulnerability in older individuals.

Our study showed that there was a large decrease in ADL and IADL disabilities in 2011/12 that later rebounded. This might be due to the impact of the economic crisis. This could also be possibly due to the fact that more older persons lived in institutions for the 2011/12 year, but our study population only included non-institutionalized individuals. Nevertheless, older people who live in institutions have more disability as compared to those who are non-institutionalized [31,32]. However, a slight decrease in disability trends from 2009 to 2017 was revealed, which were similar to studies performed in other countries (e.g., China [33] or Sweden [16]). The prevalence of IADL disabilities was higher than that of ADL disabilities, which is in agreement with previous literature [6,7,34]. It appears that despite life expectancy increasing, disability trends are slightly decreasing. This may be due to advances in medicine in recent decades and improved socioeconomic status, including a higher educational level and increased household income [26]. This decrease predicts an increase in the quality of life for older individuals.

In our study, in 2017, the ADL disability was 11.3%, while the IADL disability was 35.1%. This prevalence is important because disability status is an indicator in the prediction of adverse results and mortality risk [29]. These data are similar to findings in recent studies conducted in countries such as China [33], England [35] and the United States [36]. However, there are studies of locations with higher prevalence, such as a study conducted in Brazil [34], or with lower prevalence, such as a study conducted in the United States [6]. These discrepancies can be due to socioeconomic and cultural differences or the methodology used [8]. The prevalence rate that we have obtained is lower than previous studies conducted in Spain with non-institutionalized older people [17–19]. This difference may be because health policies in Spain are showing increasing effectiveness, and disability is therefore decreasing. However, our study shows a recent prevalence of disability with a representative population of non-institutionalized older people.

In our study, disability is associated, as in other studies, with female gender [1,8,9,14,16,33], age older than 85 years [1,8,14,33], not having higher educational status [4,14,37], habitual activity restrictions [20], being bedridden and experiencing pain [38,39]. Women are affected in greater numbers, because women not only have higher life expectancies but also have more health problems [17]. Previous studies associated age with disability, because comorbidities, health problems and disabilities increase with age [8,14]. A lower educational status is predicted to be associated with disability, because people may have problems managing money or performing other more complex cognitive functions [4,14,37]. Pain and restriction of activity can precede IADL and ADL disabilities [19,39]. However, our study is the first that associates disability status with being bedridden in the past 2 weeks. It is possible that these persons had been ill and thus could not do their activities of daily living each day. In other studies, disability is associated with rural living [28,31], depression [37] and obesity [7,35]. It appears that the profile of the disabled older person is similar in all contexts, despite the social and demographic differences in the different countries.

### Relevance to clinical practice

In Spain and Mediterranean countries, in general, older people are not institutionalized. The help that older people with disabilities need is provided in many cases by informal caregivers. It is possible for older people to improve their ability to perform ADL and/or to reduce their limitations [40]. Some interventions such as physical exercise, use of aids or devices, personal assistance and home adaptations can help to reduce disability in these individuals. Additionally, informal caregivers can postpone moving older people to institutionalized care settings, according to some studies [1]. Therefore, it is especially important to support informal caregivers, and policymakers should create programs that provide them with resources for help and assistance. For this support, the role of nurses who work in primary care is very important, because they should provide access to these older individuals, work for reducing ADL and IADL disabilities, develop appropriate interventions for maintaining their independence and encourage them to exercise regularly, as exercise is very important to maintain their functional abilities [41,42].

Early detection of disability can be beneficial for individuals who need more attention [29]; this requires functional assessments to be performed by physicians, physiotherapists and nurses in a primary care setting. Health professionals should implement geriatric evaluations regularly to identify their health care needs, with a goal to improve the overall health status of the elderly [5,43].

### Limitations

The present study has some limitations. First, the data are self-reported. Although this could be a possible bias, studies have shown that self-reported disability is congruent with disabilities diagnosed by medical services [29]. Second, we were not able to determine the causality among the related variables and disability, as the data came from a cross-sectional survey. Third, NHSS and EHSS did not collect variables that may influence disability, such as morbidity, psychological and cognitive aspects and current diseases. It is necessary to conduct additional studies to analyze temporal trends and determine the complexity level of disabilities in older individuals.

The main strength of our study is that the data came from a representative, valid and reliable Spanish survey. Therefore, it is beneficial in offering representative data about disabilities in older individuals in Spain, which may be useful for health professionals in developing preventive programs and interventions.

## Conclusions

In conclusion, this study provides valid data about disability in Spanish non-institutionalized older people. The prevalence of ADL and IADL disabilities is considerable, and almost one-third of people over 65 years old have some form of IADL disability, with IADL disabilities having a higher prevalence than ADL disabilities. ADL and IADL disabilities are associated with female gender, being older, having lower educational status, having activity restrictions, being bedridden and experiencing pain. Although the disability prevalence decreased slowly from 2009 to 2017, disability remains a health problem that is associated with negative outcomes and poorer quality of life among older people.

Therefore, it is necessary to improve prevention strategies and to establish effective interventions for reducing or reversing disability status in older people.
